# Enhancing Protein Crystal Nucleation Using In Situ Templating on Bioconjugate-Functionalized Nanoparticles and Machine Learning

**DOI:** 10.1021/acsami.2c17208

**Published:** 2023-02-28

**Authors:** Caroline McCue, Henri-Louis Girard, Kripa K. Varanasi

**Affiliations:** Department of Mechanical Engineering, Massachusetts Institute of Technology, 77 Massachusetts Avenue, Cambridge, Massachusetts 02139, United States

**Keywords:** protein crystallization, machine learning, bioconjugates, nanoparticles, nucleation, downstream processing

## Abstract

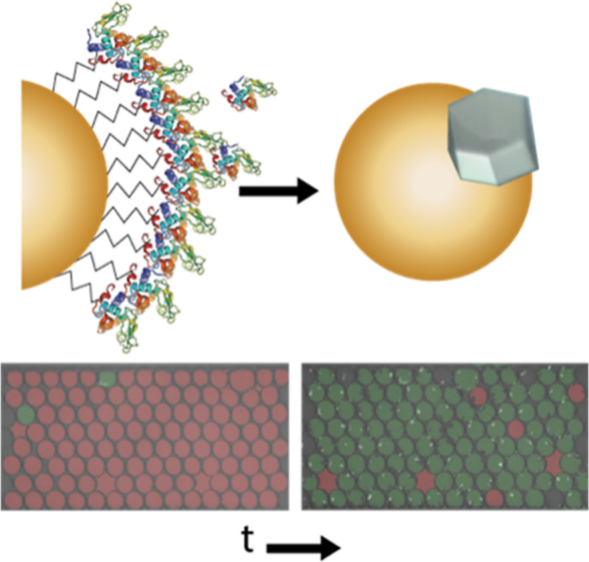

Although protein crystallization offers a promising alternative to chromatography for lower-cost protein purification, slow nucleation kinetics and high protein concentration requirements are major barriers for using crystallization as a viable strategy in downstream protein purification. Here, we demonstrate that nanoparticles functionalized with bioconjugates can result in an in situ template for inducing rapid crystallization of proteins at low protein concentration conditions. We use a microbatch crystallization setup to show that the range of successful crystallization conditions is expanded by the presence of functionalized nanoparticles. Furthermore, we use a custom machine learning-enabled emulsion crystallization setup to rigorously quantify nucleation parameters. We show that bioconjugate-functionalized nanoparticles can result in up to a 7-fold decrease in the induction time and a 3-fold increase in the nucleation rate of model proteins compared to those in control environments. We thus provide foundational insight that could enable crystallization to be used in protein manufacturing by reducing both the protein concentration and the time required to nucleate protein crystals.

## Introduction

Crystallization is the primary method for determining the structure of proteins; however, it typically requires very high protein concentrations and is a very slow process.^1,2^ Despite these challenges, there has recently been significant interest in using protein crystallization as a purification step in downstream protein manufacturing.^3−6^ Protein A chromatography, the traditional method of protein purification, relies on expensive, specialized resins and strict quality control to maximize separation efficiency and minimize impurities.^7^ As a result, separation and purification of proteins account for up to 50% of the manufacturing cost.^8,9^ However, there are still major challenges that must be addressed before protein crystallization can be used at the industrial scale. Even in optimal conditions, growing protein crystals can take days or even weeks and usually requires high protein concentrations ranging from 2 to 100 mg/mL.^1^ These required concentrations are significantly higher than bioreactor outputs, which typically range from 5 to 20 g/L.^6^ As a result, proteins must be further concentrated before they can successfully be crystallized. In addition to crystallization, there has been significant research into alternate purification processes such as cation exchange and mixed-mode chromatography, membranes, ultrafiltration, precipitation, and microfluidic devices.^10,11^ Microfluidic approaches such as liquid–liquid extraction and aqueous two-phase systems can offer another route to handling low-concentration separation. In fact, droplet-based microfluidics can even help address some protein crystallization challenges because such devices offer high mixing efficiency, high mass transfer, reduced crystallization time, and low material quantity requirements.^12^

**Figure 1:**
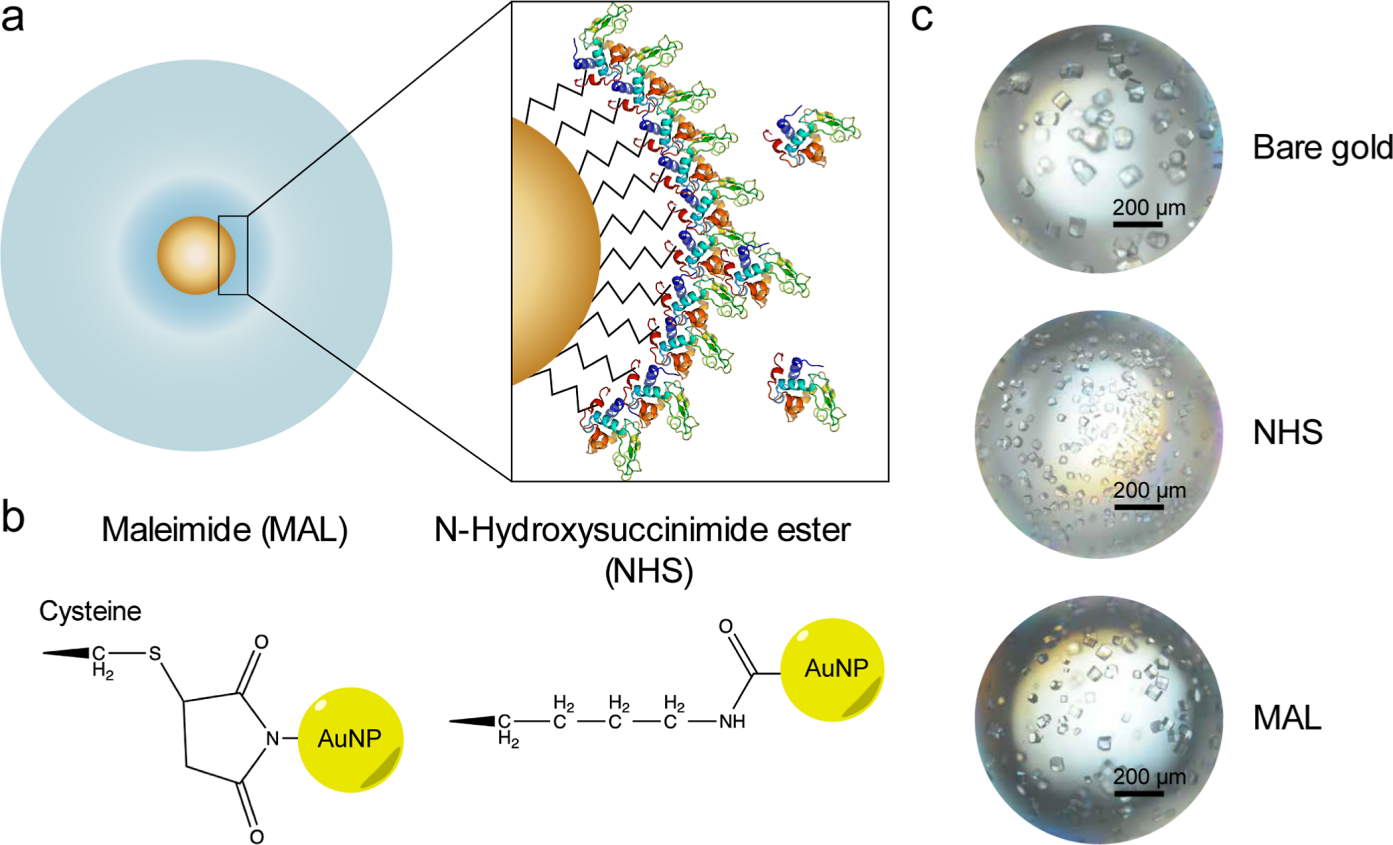
(a) Schematic illustrating the concept of nanoparticle-assisted nucleation. (b) Conjugation of proteins to gold nanoparticles via the target amino acids. (c) Optical images of representative crystals obtained from vapor diffusion experiments with different nanoparticles.

We seek to address these challenges by using bioconjugate-functionalized nanoparticles to enable nucleation of crystals in lower protein concentration conditions and increase the nucleation rate. Previous use of nanoparticles in protein crystallization typically relied on electrostatic interactions or adsorption. In contrast, our aim is to bind proteins to specific sites using a templated architecture. Our approach is illustrated in Figure 1a: Nanoparticles are functionalized to selectively form covalent bonds to specific amino acids, creating an in situ layer of proteins that act as a template for the next layer of proteins. Indeed, proteins naturally self-assemble on the nano- and microscale to form highly ordered structures such as crystals, fibrils, or amorphous aggregates.^13^ Previous work has shown that intermolecular interactions drive protein aggregation and fibrillation.^14,15^ In addition, nanofibril structures can even be designed by modifying specific bonds or amino acid residues.^16^ A surface of highly ordered proteins can drive the protein crystal nucleation in the same way that seeding a solution with small protein crystals (often practiced in large-scale crystallization) can enhance protein crystal growth. Prior studies have robustly shown that the use of nanoparticles and other surfaces can seed crystal formation through the introduction of heterogeneous nucleation sites.^17−22^ It is well-established that heterogeneous nucleation requires a lower energy barrier than homogeneous nucleation, such that the rate of nucleation near a surface is much greater than in the bulk.^23^ Gold and silica nanoparticles have been shown to increase the number of lysozyme crystals formed compared with control solutions due to increased interactions between the particles and proteins.^24−26^ The impact of surface wettability has been studied using functionalized nanoparticles, and hydrophilic nanoparticles have been shown to result in higher nucleation rates.^27^ Nanoparticles have also been used to improve the quality and size of crystals produced for X-ray diffraction studies.^28^ We aim to use functionalized nanoparticles to enhance nucleation rates and enable low concentration crystallization, thus making it viable to be used in protein purification. Encouragingly, previous research has indeed shown that proteins will crystallize even in the presence of other proteins, suggesting that crystallization as a separation technique is a viable option if other concerns such as speed and concentration are addressed.^29^

Our approach uses bioconjugate-functionalized surfaces to form a covalently bonded protein template in situ because it is kinetically favored for the same amino acid in each protein to react to the bioconjugate surface. This will thus impose the same orientation for all proteins in the template layer. The template forces alignment of proteins local to the nanoparticle, causing rapid crystallization. Bioconjugates are commonly used in protein and cell research to tag proteins and in drug delivery to attach proteins to nanoparticles but have not previously been used in protein crystallization.^30−32^ Furthermore, in situ templating has been rarely used for protein crystallization in bioprocessing, though there are some examples such as using polyoxometalates (POMs) to crystallize proteins for structural characterization.^33−35^ However, bioconjugates have advantages over POMs in terms of biocompatibility and ability to interact with most proteins. Here, we use bioconjugates, maleimide (MAL), and *N*-hydroxysuccinimide ester (NHS) as functionalizations for gold nanoparticles. Both react with specific amino acids (shown in Figure 1b)—MAL reacts with the thiol group present in cysteine and NHS reacts with the primary amine in lysine or the N-terminus of proteins. We chose to study lysozyme and insulin, two clinically relevant, well-characterized proteins which crystallize readily and whose self-assembly kinetics have been explored as a function of protein structure.^14^ In this study, we evaluate the impact of these bioconjugates on protein crystallization and examine how they affect protein crystallization kinetics.

## Experimental Methods

We demonstrate our crystallization strategy on a model protein, lysozyme, which has well-known crystallization conditions.^26,27,36,37^ We conducted three sets of crystallization experiments to characterize our approach: vapor diffusion, batch crystallization, and droplet crystallization. Vapor diffusion was used to qualitatively screen the crystallization outcomes for crystal size, quality, and quantity. Batch crystallization was used to measure protein crystallization probability across a range of protein and salt concentrations. Finally, droplet crystallization was used to measure the nucleation rate and induction times of crystallization.

### Vapor Diffusion

Vapor diffusion is a technique commonly used to screen crystallization conditions, in which a sitting or hanging drop of protein solution and precipitants is sealed within an environment containing a reservoir solution with a larger volume and higher precipitant concentration. The transfer of water from the drop to the reservoir as the solution equilibrates drives protein saturation within the drop and initiates nucleation.^2^ For these experiments, we used hen egg white lysozyme (MilliporeSigma) at a concentration of 20 mg/mL and sodium chloride at a concentration of 30 mg/mL in a 50 mM sodium acetate buffer at pH 4.5.^36^ For each experimental condition, 16 μL of nanoparticles (OD 1, concentration 5 × 10^13^ particles/mL) were added to 1 mL of solution. 3 μL droplets of these solutions and the control were placed in vapor diffusion plates with reservoirs of 100 mM sodium acetate and 60 mg/mL sodium chloride and then sealed. The droplets were imaged with an optical microscope (Zeiss AxioZoom) after 20 h at room temperature.

### Batch Crystallization

In batch crystallization, bulk protein solution is mixed with precipitants and then sealed to prevent evaporation. The samples were kept in sealed PCR tubes for the duration of the crystallization phase. Aliquots from these reaction tubes were taken for visualization and imaging at the end in the form of droplets. Unlike with vapor diffusion, the crystallization conditions remain constant throughout the trial. A high lysozyme concentration (50 mg/mL) solution and a high sodium chloride concentration (150 mg/mL) solution were prepared. A 0.2 μm syringe filter was used to remove any undissolved or aggregated proteins from the lysozyme solution. The protein and salt solutions were mixed just before nanoparticles were added to achieve the target concentrations and supersaturation levels in each condition. 24 μL of functionalized nanoparticles were then added to each 1.5 mL batch (OD 1, concentration 5 × 10^13^ particles/mL) except for the control case. Each batch was divided into eight Eppendorf tubes (150 μL) and sealed. After 72 h at room temperature, a 30 μL droplet was withdrawn from the bottom of each tube, placed on a microscope slide, and imaged with an optical microscope (Zeiss AxioZoom).

### Microfluidic Chip Fabrication

The droplet generator design was prepared using AutoCAD and printed on a high-resolution transparency mask (CAD/Art Services, Inc). Standard soft lithography techniques were used to produce masters of the microfluidic design. In brief, a 200 μm layer of SU-8 2100 (MicroChem) was spin-coated onto a silicon wafer, a mask aligner (Electronic Visions 620) was used to expose and cross-link the design through the transparency mask, and then the unexposed photoresist was removed using propylene glycol monomethyl ether acetate. Masters with the droplet generator design were then functionalized using 1*H*,1*H*,2*H*,2*H*-perfluorododecyltrichlorosilane (Sigma, 729965) to render the design hydrophobic by depositing 50 μL onto a glass slide and placing alongside the master under vacuum for 2 h. To produce the microfluidic device, polydimethylsiloxane (PDMS) (Sylgard 184, Dow Chemical) was mixed according to package directions, poured onto the master, degassed, and cured at 75 °C for 1 h. The PDMS microfluidic device was then peeled away from the master, trimmed, and then oxygen plasma-treated (Glow Plasma Systems) along with a 50 × 75 mm glass slide for 2 min. Immediately upon removal from the plasma cleaner, the PDMS device was pressed onto the glass slide to bond it. The insides of the droplet generator devices were rendered hydrophobic by flowing AquaPel, followed by air, through the channels to ensure that the oil phase wets the PDMS rather than the aqueous phase.

### Microfluidic Platform

To generate a population of identical, independent droplets, we developed a microfluidic platform combining a microfluidic mixer and an emulsion generator. The two inner inlets enabled the separate introduction of an undersaturated solution of proteins and precipitant salts/nanoparticles. These streams were then mixed on-chip at a junction before the droplet generator, ensuring that the protein solution becomes supersaturated at a controlled moment and as late as possible in its preparation. The next stage was a junction droplet generator that created identical droplets by pinching the flow of the protein solution with a flow of biocompatible fluorinated oil: HFE7500 + 2% 008-FluoroSurfactant (Ran Biotechnologies).^38^ Each of these inlets was connected to a pressure vessel which was in turn connected to a pressure controller (Fluigent Flow EZ). The switchbacks after each inlet were flow resistance devices to help prevent backflow. For each experiment, the pressure vessels were filled with the appropriate protein and precipitant streams, and then the pressure of these inlets was adjusted so that their flow rates were equal and the pressure of the oil inlet was increased until the transition from jetting to droplet formation occurred. Once a stable stream of identical droplets was generated, a thin rectangular capillary was brought in contact with the outlet of the microfluidic chip and the emulsion was drawn inside by capillary forces. The thickness of the capillary, 200 μm, was such that the droplets arranged in a single layer. The other tube dimensions, 2 × 100 mm, were chosen to facilitate imaging and maximize the number of droplets visible. The tube was then sealed with a mix of lanolin, vaseline, and paraffin wax to prevent evaporation.^39^ Finally, a microscope connected to a camera was used to image the emulsion at regular time intervals (once per minute). Two polarizers were installed at right angles to each other along the light path before and after the capillary, such that protein crystals appear bright in the resulting images, enabling easier identification of crystals in the images using the machine learning algorithm.

### Droplet Crystallization

A concentration of lysozyme of 40 mg/mL in a sodium acetate buffer (50 mM, pH 4.5) was used in the protein stream, and 120 mg/mL of NaCl in sodium acetate buffer (50 mM, pH 4.5), which contained the nanoparticles, was used as the precipitant stream.^36^ These streams were mixed within the microfluidic chip for a final concentration of 20 mg/mL lysozyme and 60 mg/mL NaCl in sodium acetate buffer within the emulsions.

### Insulin Crystallization

Insulin experiments were performed following the same protocols as the lysozyme experiments. The crystallization conditions for the vapor diffusion experiments were 2.5 mg/mL recombinant human insulin (MilliporeSigma) dissolved in 50 mM citrate buffer at pH 6.5 and addition of 20 mM ZnCl_2_. For the nucleation rate experiments, 2.5 mg/mL insulin dissolved in 50 mM citrate buffer at pH 6.5, 5 mM ZnCl_2_, and 10% acetone were used.^40^

### Nanoparticles

5 nm gold nanoparticles with bioconjugations (Cytodiagnostics) were purchased from MilliporeSigma (MAL: SKU 900458 and NHS: SKU 900470). The lyophilized, ready-to-use nanoparticles were rehydrated using the resuspension buffer supplied to a volume of 100 μL and then diluted to OD 1 using the appropriate buffer for each protein and used immediately. At OD 1, the peak SPR wavelength is 515–520 nm, the size dispersity is <15%, the nanoparticle concentration is 5.47 × 10^13^ particles/mL, the weight concentration is 6.94 × 10^–2^ mg/mL, the particle volume is 65.4 nm^3^, the particle surface area is 78.5 nm^2^, the surface/volume ratio is 1.2, the particle mass is 1.27 × 10^–18^ g, and the molar extinction coefficient is 1.1 × 10^7^ M^–1^ cm^–1^.

### Statistical Analysis

**Figure 2:**
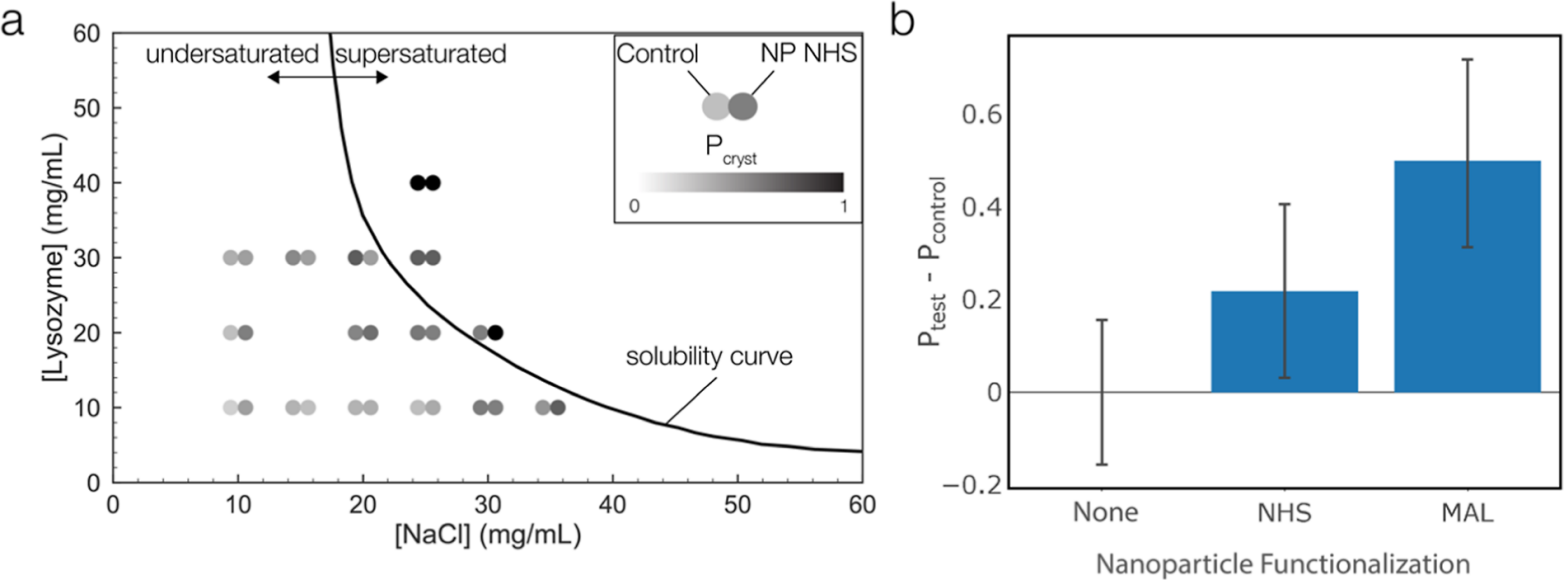
(a) Nucleation probability after 72 h, where each pair of circles shows the nucleation probability for the control case on the left and with NHS-functionalized nanoparticles added on the right for each crystallization condition on the solubility diagram of lysozyme. The darker shaded circles signify a higher nucleation probability. (b) Increase in crystallization probability relative to the control (no nanoparticles) for each nanoparticle functionalization. Error bars represent 95% confidence intervals (*n* = 32).

For the batch crystallization analysis, data shown in Figure 2a are the percent of trials (*n* = 32), which displayed crystals. Figure 2b shows the difference in the percent of samples showing crystals between each of the nanoparticle experiments and the control and uses bootstrap analysis to determine 95% confidence intervals. For the emulsion crystallization analysis, we plotted the fraction of (number of clear drops)/(number of drops) at each time point from the beginning of the experiment (at *t* = 0) until *f*_clear_ = 0. For lysozyme results, the induction time and the nucleation rate data shown are the mean ± SD of trials performed (*n* = 3). For insulin results, they are from one set of droplets, and error bars show standard error associated with the machine learning algorithm. Induction time was calculated at *f*_clear_ = 0.75, and nucleation rate was calculated from the data within the range of 0.1 > *f*_clear_ > 0.75. For all experiments, data and statistical analyses were performed using matplotlib tools in python.

## Results

### Vapor Diffusion

Representative outcomes of the vapor diffusion experiments are shown in Figure 1c for bare gold nanoparticles and NHS- and MAL-functionalized gold nanoparticles. We observed a dichotomy where the bare gold nanoparticles led to fewer, larger crystals, while the bioconjugate-functionalized nanoparticles led to a larger number of small crystals. This first result is consistent with prior protein crystallization literature using nanoparticles.^21,28,41,42^ These previous studies report that the addition of nanoparticles can result in larger crystals which are favorable for use in X-ray diffraction. In contrast, our results on NHS- and MAL-functionalized nanoparticles show a larger number of smaller crystals of lysozyme, which is indicative of a higher nucleation rate.^43,44^

Vapor diffusion experiments allow us to visualize qualitatively the differences in protein crystallization outcomes; however, they do not provide information on the crystallization process. For instance, because both protein and salt concentrations are changing throughout the experiment, it is difficult to identify nucleation rates or the minimum concentration for nucleation. Therefore, to develop a clearer understanding of the impacts of supersaturation, we used batch crystallization.

### Low-Concentration Crystallization

Batch crystallization experiments (see Methods) were used to measure the nucleation of the lysozyme in undersaturated conditions. We probed a range of crystallization conditions at different saturation levels by varying the sodium chloride concentration based upon the known solubility curve for the lysozyme (Figure 2a).^36,45^ The spectrum of possible outcomes included clear droplets, droplets containing crystals, or droplets showing aggregated and precipitated protein.

We observed the outcome of crystallization for each drop and then calculated the nucleation probability as the ratio of the number of drops containing crystals to the total number of drops observed. Drops containing precipitated proteins were omitted from the analysis. In Figure 2a, each pair of circles represents the nucleation probability for the control case (with no nanoparticles added) on the left and the nucleation probability in the case with NHS-functionalized nanoparticles added on the right at each crystallization condition. The darker shaded circles represent a higher nucleation probability. As expected, in conditions of high supersaturation, there was a high nucleation probability in both the control case and the nanoparticle case (e.g., [Lyz] = 40 mg/mL, [NaCl] = 25 mg/mL). Interestingly, in the case of low supersaturation ([Lyz] = 20 mg/mL, [NaCl] = 30 mg/mL), the nanoparticle addition increased the nucleation probability by 30%.

To examine the industrially relevant case of low protein concentration, we further tested the crystallization at a low protein concentration at a point below the saturation curve ([Lyz] = 10 mg/mL, [NaCl] = 35 mg/mL).^6^Figure 2b shows the results of additional batch crystallization experiments (32 replicates each). Here, each bar shows the difference between the nucleation probability in the test case (with nanoparticles) and the control case (with no nanoparticles added). These results show that bare nanoparticles did not have a significant effect on nucleation probability, while the addition of NHS- and MAL-functionalized nanoparticles led to 20 and 50% increases in nucleation probability compared with the control. These results indicate that in situ templating via bioconjugate-functionalized nanoparticle surfaces can enable nucleation in low protein-concentration solutions that would otherwise be undersaturated.

### Nucleation Kinetics

**Figure 3:**
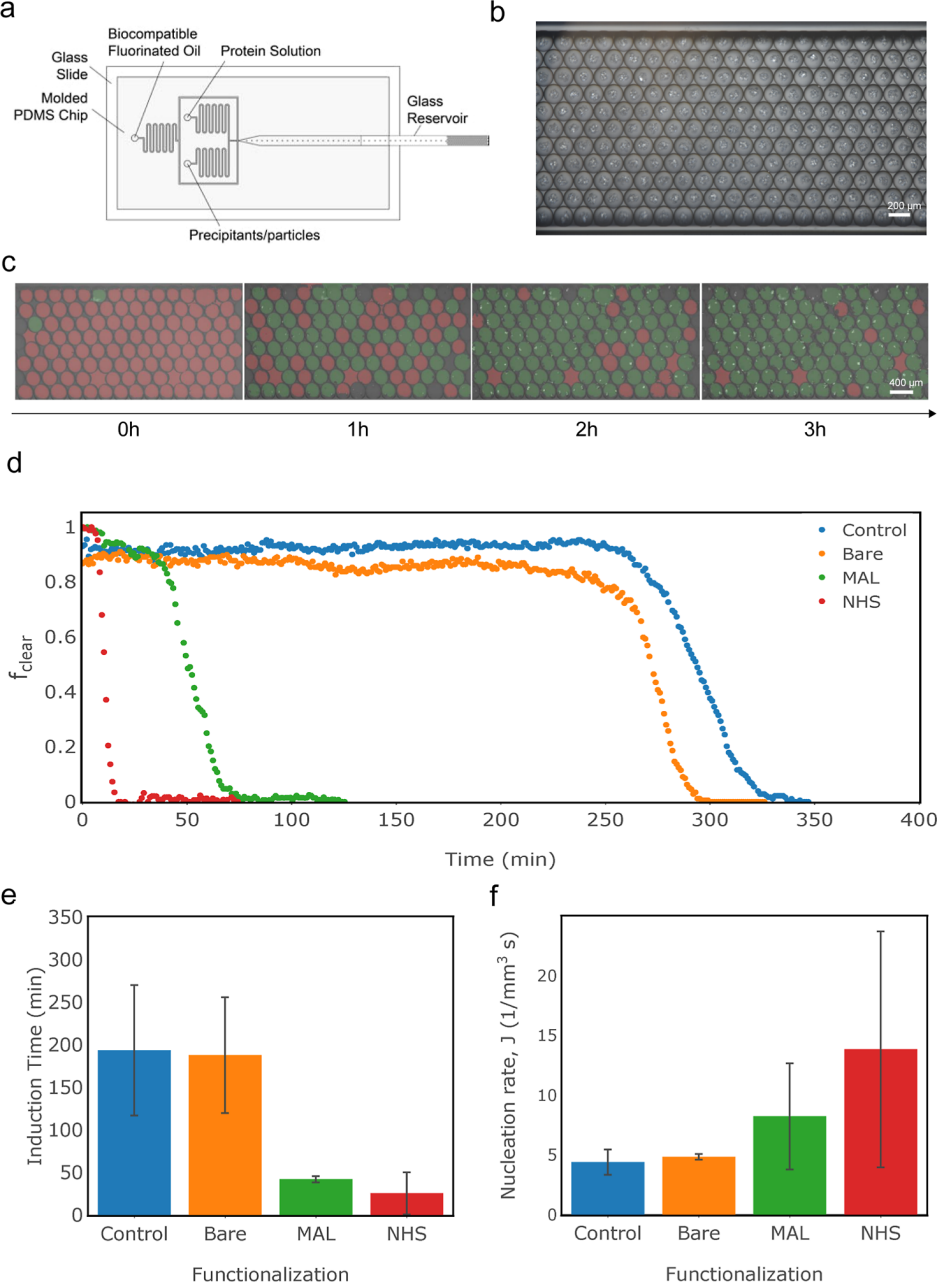
Lysozyme crystallization; (a) schematic of the microfluidic droplet generator, (b) raw optical image of lysozyme crystals within emulsion droplets at the end of a nucleation experiment, (c) chronophotography images with red/green overlay to indicate droplets containing crystals, (d) fraction of clear droplets as a function of time for each condition (plot shows data from one experiment), (e) induction time, and (f) nucleation rates for lysozyme crystallization as a function of nanoparticle functionalization (bar graphs show average of three sets of droplets; error bars show standard deviation).

Nucleation of protein crystals is a stochastic phenomenon; thus, we must measure a large population in order to determine the nucleation rate.^46^ To do so, we used a microfluidic droplet generator to produce a large quantity of identical, independent droplets within which the proteins were crystallized (see Experimental Methods).^2,47,48^ A schematic of the device used to produce the emulsions containing the protein and precipitants is shown in Figure 3a (see the Supporting Information for a schematic of the platform and images of the microfluidic device). Capillary tubes were used to collect the emulsions and sealed to prevent evaporation. The emulsion drops were imaged once per minute until protein crystals were observed in all drops (Figure 3b).

Due to the large number of images, each containing hundreds of drops, we developed a custom machine learning approach to automate image processing and analyze the data. Our algorithm segments the initial image, isolates each droplet, and classifies images based on the presence of crystals in a given droplet. The overall machine learning approach is described in the Supporting Information. A representative time-series in Figure 3c shows individual images at different time points with colors overlaid to indicate clear drops (red) and drops containing crystals (green). Figure 3d shows a comparison between each of the nanoparticle conditions of the fraction of clear droplets, *f*_clear_, over time (see Methods for experimental conditions). From these graphs, we can determine important crystallization parameters of induction time and nucleation rate. The induction time typically refers to the delay between the onset of supersaturation and the visible appearance of crystals, and the nucleation rate is taken as the number of nuclei that form per unit volume as a function of time. Qualitatively, in the control case without any nanoparticles added, we observe that it takes longer before crystals begin to appear and that the rate of crystals appearing is slower compared with the MALand NHS cases. All of the conditions exhibit a response with the same general sigmoidal shape of the fraction of clear drops over time, as we would expect to observe.

For nucleation rate *J*, at a particular supersaturation, the probability of crystal formation within a droplet of volume *V* during time interval d*t* is *P*_crystal_ = *JV* d*t*. In a population of *N* identical droplets, the fraction *f*_clear_ = *N*_clear_/*N* of droplets within which a crystal has not nucleated is equal to the probability *P*_clear_(*t*) that a single droplet has remained clear until time *t* and follows a usual exponential decay pattern such that^46,47^
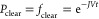
1

Induction time and nucleation rate were evaluated by measuring *f*_clear_ across a population of drops. Consistent with previous studies, we define induction time as the amount of time taken for the first visible crystals to appear from the point at which the capillary tubes are sealed.^49^ This time is quantitatively extracted from *f*_clear_(*t*) data (e.g., Figure 3d) as the time at which 25% of the droplets contain crystals. This threshold was chosen to robustly classify droplets with crystals, as opposed to experimental artifacts such as debris on the capillary tube. The induction time results confirm that the addition of functionalized nanoparticles significantly reduces the amount of time taken before crystals begin to appear compared to both the control (with no nanoparticles added) and the addition of bare gold nanoparticles (see Figure 3e). Compared with the control, the addition of functionalized nanoparticles reduced the induction time by an average factor of 4.5 for MAL and 7.5 for NHS, while the addition of bare gold nanoparticles did not significantly decrease the induction time. These results illustrate that the use of bioconjugate-functionalized nanoparticles influences nucleation more than simply the addition of heterogeneous nucleation sites.

The nucleation rate was derived by linearizing and fitting the exponential decay portion of a semilog plot of the data shown in Figure 3c (see the Supporting Information). Nucleation rate is calculated from the data within the range of 0.1 > *f*_clear_ > 0.75. As shown in Figure 3f, the control case resulted in the smallest average nucleation rate of 4.4 mm^–3^ s^–1^, and the bare nanoparticles did not show any appreciable change in the nucleation rate despite a relatively long induction time. In contrast, the MAL- and NHS-functionalized nanoparticles increased the nucleation rates to an average of 8.2 and 13.8 mm^–3^ s^–1^, respectively.

### Circular Dichroism Measurements

After crystallization, we collected crystals, redissolved the crystals in fresh buffer, and measured the fluorescence and circular dichroism (CD) spectra of the proteins (see Supporting Information). We saw no significant differences in the fluorescence or CD spectra for proteins that had been crystallized in the presence of any of the gold nanoparticles compared with the control, suggesting that the use of functionalized gold nanoparticles has no lasting effect on the secondary structure of the redissolved proteins.

### Insulin Crystallization

**Figure 4:**
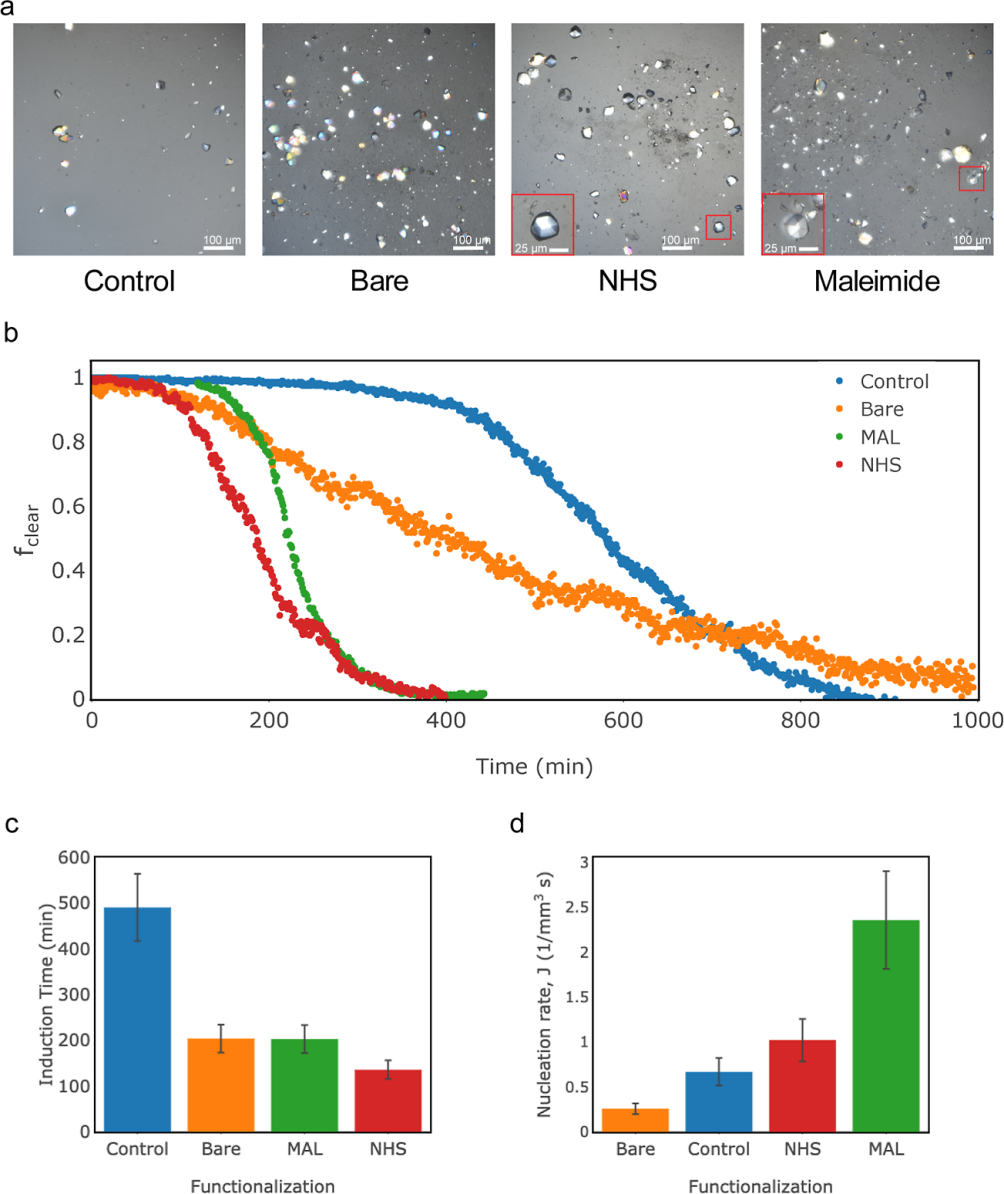
Insulin crystallization. (a) Representative vapor diffusion results, with insets showing magnified crystals, (b) Fraction of clear droplets as a function of time for each condition, (c) induction time, and (d) nucleation rate for insulin crystallization as a function of nanoparticle functionalization (bar graphs show the results of one set of droplets, and error bars show standard error from the machine learning algorithm).

Our functionalized nanoparticle approach can be extended to other proteins. We tested the crystallization of human insulin using vapor diffusion and droplet approaches. Typically, insulin crystallization requires high concentration of insulin (5–7 mg/mL) and precipitants.^50,51^ Our vapor diffusion experiments confirm that the bioconjugate-functionalized nanoparticles can crystallize insulin at lower concentrations (2.5 mg/mL) that are consistent with industrially relevant insulin bioreactor outputs (typical yields range from less than 1 mg/mL up to 4 mg/mL).^52^ We followed the same experimental procedures as with lysozyme to examine the effects of the functionalized nanoparticles on insulin crystallization. We saw similar vapor diffusion results as with lysozyme; the addition of functionalized nanoparticles resulted in a larger number of smaller crystals compared with the control, while bare nanoparticles resulted in fewer, larger crystals (Figure 4a).

We then conducted droplet crystallization experiments to obtain the induction time and nucleation rate for insulin (Figure 4b–d). As shown in Figure 4b, the functionalized nanoparticles demonstrate a higher nucleation rate when compared to control and even bare nanoparticles. The induction times were significantly lower for the experiments where nanoparticles were present; however, unlike with lysozyme, the case of the bare nanoparticles resulted in an induction time similar to the case of the MAL-functionalized nanoparticles, while the induction time in the case of the NHS-functionalized nanoparticles was only slightly shorter (Figure 4c). Similar to lysozyme, the addition of functionalized nanoparticles demonstrated 50–250% higher nucleation rates than the control (Figure 4d).

## Discussion

The addition of bioconjugate-functionalized nanoparticles resulted in both lower induction times and higher nucleation rates, an optimal combination for reducing the time it takes to crystallize a product of interest in the context of protein purification. The induction time and nucleation rate trends for both lysozyme and insulin are very similar, with the bioconjugate-functionalized nanoparticles demonstrating the highest nucleation rates and the lowest induction times in both cases. However, the comparison with the bare gold nanoparticles is not as clear. Unlike in the case of lysozyme, for insulin, the bare gold nanoparticles show the slowest nucleation rate but a shorter induction time. In the absence of a catalyzing factor, the induction time is a stochastic process. The protein-specific differences in the nucleation rate arise because the thermodynamics of binding of each protein to the bioconjugate depends on the accessibility of different amino acids at the surface, which is unique to each protein. In the case of the bioconjugate surfaces, protein binding is directed by the presence of the functional groups, while binding on the bare gold surfaces is driven by electrostatic interactions, which can lead to more stochastic behavior.

Previous researchers have studied how factors such as the addition of nanoparticles or the application of external forces affect the induction time and nucleation rate of lysozyme crystallization. A study on how the size of silica nanoparticles impacts lysozyme nucleation showed a 4.8-fold reduction in the induction time using 200 nm particles.^26^ A study using ultrasonic waves to enhance lysozyme crystallization showed a 2-fold decrease in the crystallization time (to 35% yield).^53^ In another study, where the nucleation rate was measured by the number of crystals formed in a sample, application of electric fields of 10 kHz on lysozyme crystallization with NaCl resulted in a decrease in nucleation rate, while application of higher electric fields of 500 kHz resulted in no change in nucleation rate compared with the control.^54^ In comparison to these previous studies, our lysozyme crystallization results for both nucleation rate and induction time are extremely promising.

## Conclusions

In this work, we demonstrate that bioconjugate-functionalized nanoparticles can significantly enhance the nucleation rate and lower the induction time for both lysozyme and insulin. From the vapor diffusion experiments, we observed that the addition of bioconjugate-functionalized nanoparticles resulted in a greater number of crystals, suggesting an increased nucleation rate. In the batch crystallization experiments, the increased probability of crystal formation in low protein concentration conditions suggests that bioconjugate-functionalized nanoparticles indeed act as templates for nucleation. Through the droplet crystallization experiments, quantitative determination of the nucleation rate demonstrated that the addition of functionalized nanoparticles resulted in a 7-fold decrease in the induction time and a 3-fold increase in the nucleation rate of lysozyme. The insulin results were similar to that of lysozyme and support the use of bioconjugate-functionalized nanoparticles for improving the nucleation rates and reducing the induction times for protein crystallization.

Furthermore, our approach could be combined with existing approaches to enhance protein crystallization that modify bulk properties, such as temperature or concentration. Bioconjugate-functionalized nanoparticles can be incorporated into existing industrial protein crystallization workflows, such as for insulin, and used to expand the viability of crystallization as a downstream purification step for new proteins. Bioconjugate functionalized nanoparticles could even be integrated into existing microfluidic devices for separation and purification. Future studies are needed to examine the effects of these nanoparticles on crystallization of proteins in mixed solutions and to implement the use of such nanoparticle seeds into current crystallizer workflows. The use of these bioconjugate-functionalized nanoparticles could enable protein crystallization as a purification method and lower the cost of downstream processing, which could lead to a reduction in the cost of crucial biologic drugs.

## Supporting Information Available

Additional experimental details, materials, and methods, including a description of the machine learning algorithm (PDF)
Emulsion being formed on the microfluidic chip (MOV)
Protein crystallization time lapse of emulsions containing the control solution (MOV)
Protein crystallization time lapse of emulsions containing the NHS-functionalized nanoparticles (MOV)

## Supplementary Material

am2c17208_si_001.pdf

am2c17208_si_002.mov

am2c17208_si_003.mov

am2c17208_si_004.mov
