# Supplementary material for: Enhancing Protein Crystal Nucleation Using In Situ Templating on Bioconjugate-Functionalized Nanoparticles and Machine Learning
Source: ACS Appl Mater Interfaces. 2023 Feb 28;15(10):12622–30. doi: 10.1021/acsami.2c17208 (PMC10020963; doi:10.1021/acsami.2c17208)
Supplement: Supplementary file 1 — am2c17208_si_001.pdf [file am2c17208_si_001.pdf]

Supporting Information

Enhancing protein crystal nucleation using in situ templating on bioconjugate-functionalized nanoparticles and machine learning

*Caroline McCue, Henri-Louis Girard, and Kripa K. Varanasi**

Department of Mechanical Engineering, Massachusetts Institute of Technology, Cambridge, Massachusetts 02139, United States

*Corresponding author: varanasi@mit.edu

S1. Microfluidic Emulsion Platform

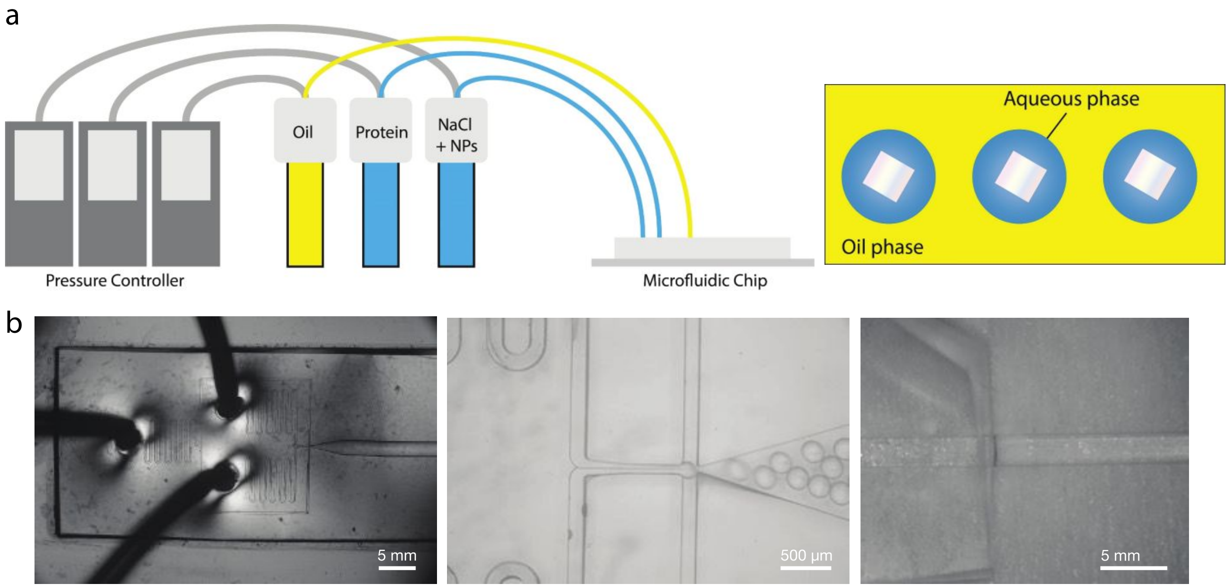

Figure S1. (a) Schematic of the microfluidic pressure-controlled platform used to produce identical droplets for nucleation experiments, and (b) images of the microfluidic chips used to make the emulsions, the streams of protein solution and precipitant combining on the chip, and the emulsions being loaded into capillary tubes.

S2. Machine Learning

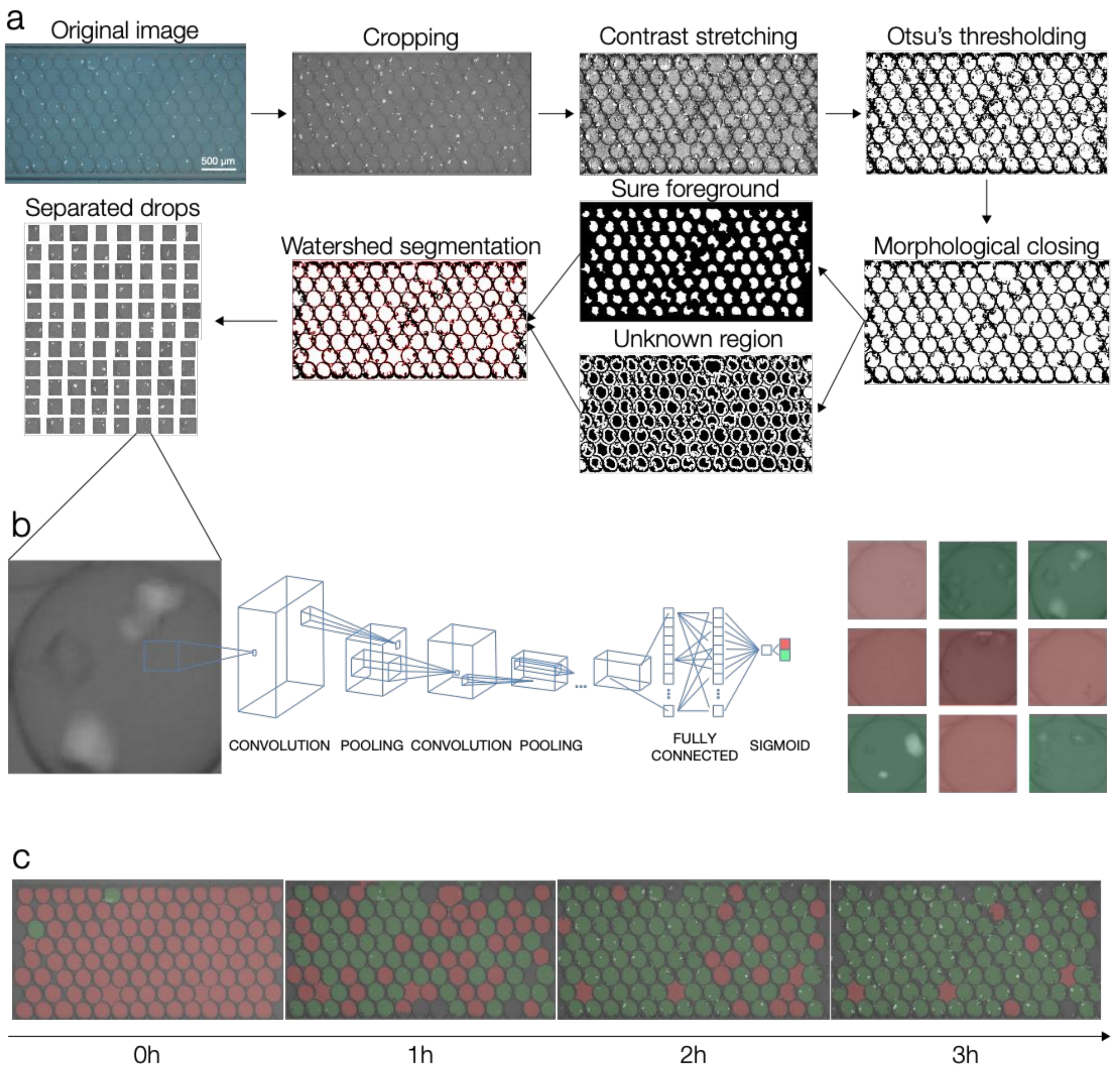

Figure S2. (a) Workflow of the segmentation algorithm, (b) schematic showing the layers of the image classification algorithm, and (c) images overlaid with the output of image classification, showing images containing a crystal in green and clear in red.

The original image is first cropped to keep only the area with droplets based on a mask provided by the user for one representative image of the experiment. A local contrast stretching algorithm

is then applied to increase the contrast of the droplet contours and compensate brightness disparities due to non-uniform illumination at the edges. The resulting grayscale image is then converted to a binary image by using a threshold value determined by Otsu's thresholding algorithm, which minimizes the intra-class variance of the intensity of the pixels assigned to the background and foreground, respectively. The resulting image generally shows a clear outline of the drops but also a number of artifacts due to the presence of the crystals. To remove these and clean up the contours, a morphological closing operation is performed where the white foreground is dilated and then contracted which smooths the outlines and erases small black spots. This is followed by an additional cleaning operation where all black continuous areas are measured and regions smaller than a specified threshold are removed. These operations result in a cleaner image where the drop outlines are well characterized. However, because of the presence of crystals at the edges of the droplets, the cleaning operations can result in multiple droplets being connected by bright pixels. This imposes the use of a segmentation algorithm that can separate these drops (**Figure S2a**). First, a distance transform is applied, where the distance of each white pixel to the nearest black pixel is converted into a grayscale value. From this intermediate image, all pixels that are farther from the background than 20% of the diameter of

the drop are considered *sure foreground* while all black pixels are considered *sure background*. A watershed segmentation algorithm is then applied to the morphologically closed image by using the *sure foreground* as seeds. This algorithm works by expanding an area around each seed pixel by pixel until it meets either the background or another expanding area (originating from another seed). It then draws a boundary around each original seed corresponding to where the expansion has stopped. Finally, each region is analyzed to eliminate artifacts based on the circularity and total area. The remaining regions correspond to droplets that are now separated.

Once the drops are separated, the presence of crystals within each one must be determined. While this task is relatively straightforward for a person, it presents a significant coding challenge due to the different forms the crystals can adopt. Indeed, their shape can vary significantly, their size increases with time during an experiment and their brightness depends on their orientation with regards to the polarized light. Because of these challenges, a traditional classification approach based on contrast detection or histogram analysis alone was insufficient to achieve an acceptable classification accuracy, thus, it was necessary to build a convolutional neural network (CNN) for this task (**Figure S2b**). This CNN was built from three convolutional

layers which reduce an area the size of the specified kernel to a single value in the next layer enabling significant dimensionality reduction while retaining spatial information. Each of these layers used a square 3x3 kernel on the 150x150 input image with no striding and a rectified linear unit (ReLU) activation. Between each of these layers a MaxPooling step with a kernel size of 2x2 was used to further down-sample the input and reduce noise. A single fully connected layer was then introduced to combine the features before the output layer which used a sigmoid activation.

To train the CNN, the segmentation algorithm described above was used to generate approximately 4,000 images of individual droplets which were manually classified as either containing crystals or being clear. The training set was built to be representative of the different type of crystals that could be encountered and balanced with close to 2,000 images in each class. Once trained over 50 epochs, the model reached about 90% accuracy on both the training and validation datasets with limited overfitting. Different versions of the model with accuracies ranging from 70 to 95% (the latter obtained by overfitting a particular subset of images) were

tested in the full implementation of the package and the fraction of clear droplets f_{clear} was found to have very little sensitivity to the accuracy of the model over 85%.

S3. Nucleation Rate Fitting

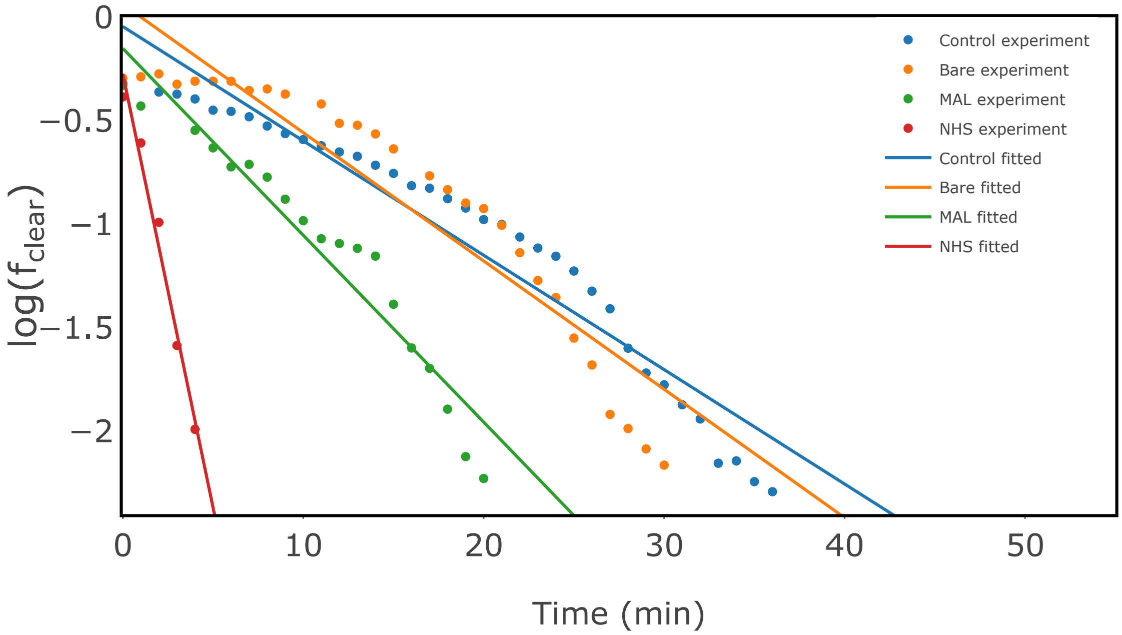

Figure S3. Semi-log plot of exponential decay for each of the nanoparticle conditions tested and their linearized fits.

To determine the nucleation rates, we linearized the exponential decay of the fraction of clear drops in a semi-log plot for each nanoparticle condition. The data was trimmed to exclude points up until the first 25% of crystals formed, in order to eliminate experimental artifacts such as

debris on the outside of the glass capillaries which would be illuminated under the polarized light, and to exclude points after 90% of crystals had formed, so as only to capture the exponential decay portion of the plot. The slope of this linearized data was used to determine the nucleation rate of the protein for each of the nanoparticle functionalizations. Assuming up to 15% error in the measurement of f_{clear}, the maximum observed from our machine learning algorithm, the estimated error for the nucleation rate measurement, based off of a simple propagation of error in Equation 1, would be 23.07%. Even with this level of uncertainty, the nucleation rate of NHS would still be approximately two times that of the nucleation rate in the control case.

S4. Lysozyme Redissolution

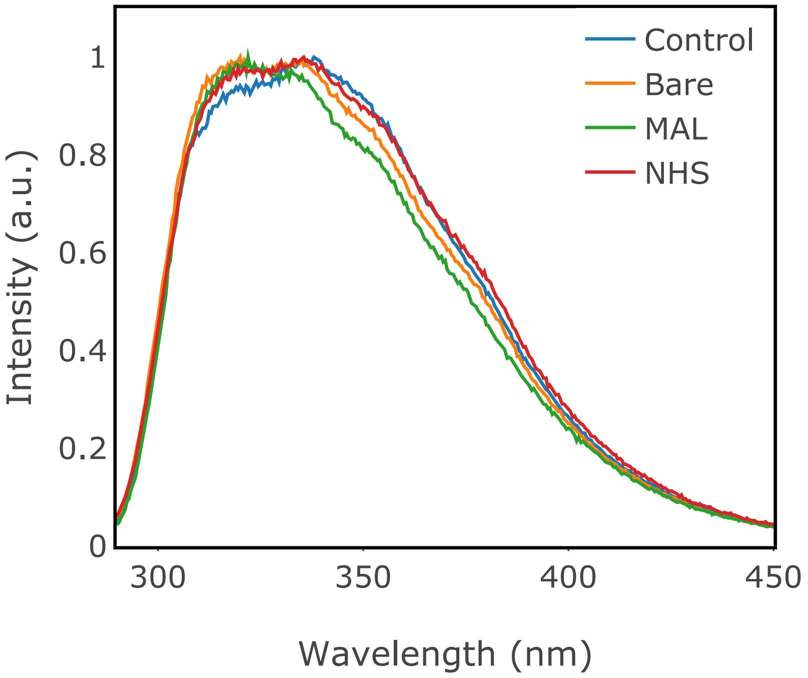

Figure S4. Fluorescence intensity over a range of wavelengths as a function of nanoparticle functionalization.

Emulsions were collected, centrifuged and separated, and lysozyme crystals were collected, washed, and redissolved following microfluidic crystallization experiments (using the same crystallization conditions). The fluorescence spectra were measured for a solution of protein that was crystallized using each of the different nanoparticles and control. No significant differences were observed in fluorescence intensity for any of the conditions, indicating that the use of bioconjugate-functionalized nanoparticles does not affect the structure of the protein upon redissolution.

Lysozyme was also batch crystallized using the same conditions as in the emulsion crystallization experiments, and crystals were collected and redissolved in DI water to perform Circular Dichroism (CD) measurements. Samples were diluted to 0.25mg/mL, and CD spectra were collected from samples at 25°C in a 1 mm quartz cuvette. No significant differences were observed in the CD spectra for any of the conditions, further suggesting that the use of bioconjugate-functionalized nanoparticles does not change the structure of the protein.

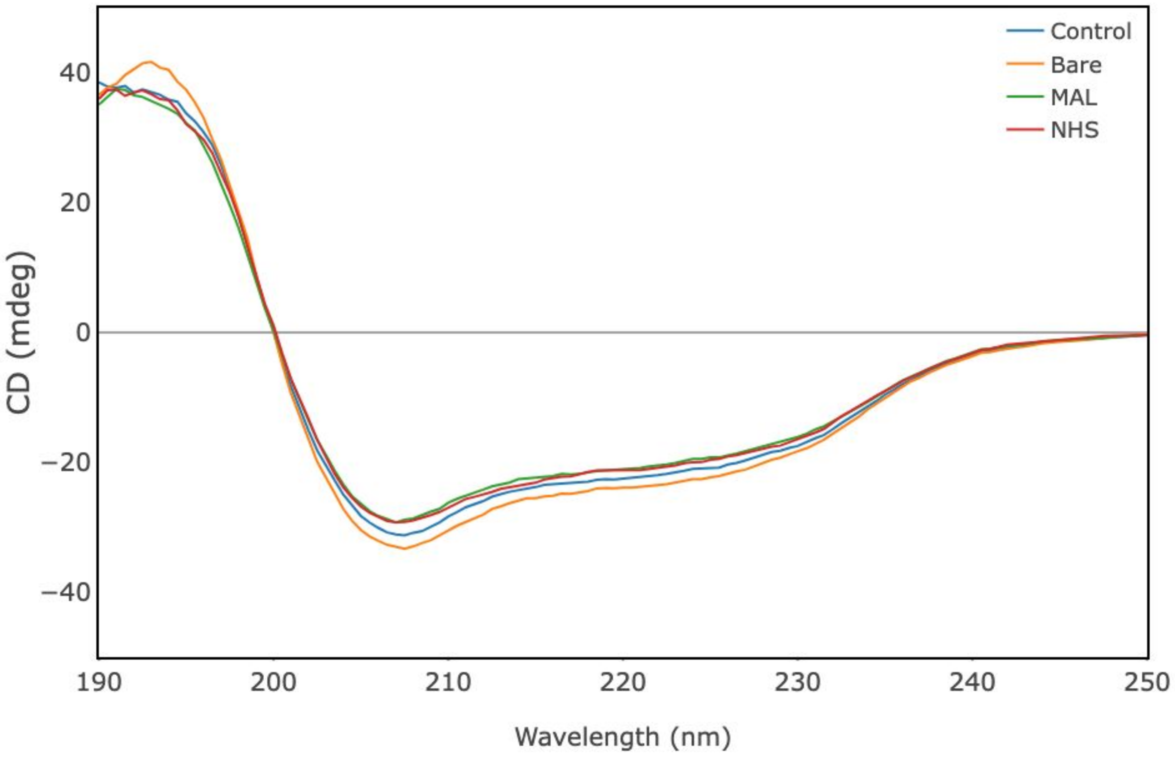

Figure S5. CD spectra from 190-250 nm as a function of nanoparticle functionalization (each plot shown is the average of 3 measurements).

Video S1. Emulsion being formed on the microfluidic chip.

Video S2. Protein crystallization time lapse of emulsions containing the control solutions.

Video S3. Protein crystallization time lapse of emulsions containing NHS-functionalized nanoparticles.
